# Medical students’ knowledge, attitudes, and practices toward generative artificial intelligence in Egypt 2024: a Cross-Sectional study

**DOI:** 10.1186/s12909-025-07329-x

**Published:** 2025-05-28

**Authors:** Omar A. Ghanem, Abdelmonam M. Hagag, Muhammed E. Kormod, Mennatullah A. El-Refaay, Alaa Mahmoud Khedr, Omnia M. Abozaid, Karim M. Abdelmoaty, Mona S. Hamed

**Affiliations:** 1 Faculty of Medicine, Zagazig University, Zagazig, Egypt; 2 Faculty of Medicine, Al-Azhar University, Madinat Nasr, Egypt; 3 Faculty of Medicine, October 6 University, 6Th of October, Giza, Egypt; 4 Faculty of Medicine, Minya University, Minya, Egypt; 5 Community Medicine Department, Faculty of Medicine, Zagazig University, Zagazig, Egypt

**Keywords:** Generative AI, Knowledge, Attitudes, Practices, Medical students, Artificial intelligence

## Abstract

**Background:**

Generative artificial intelligence (generative AI) has revolutionized the healthcare system for medical students and their educational process worldwide. Several studies assessed its efficacy in improving medical studies learning, and others assessed medical students’ knowledge and attitudes about generative AI. So, we aim to determine the knowledge, attitudes, and practices of generative AI among medical students in Egypt.

**Methods:**

In 2024, a cross-sectional study using a semi-structured questionnaire was performed, targeting 423 students as a convenient sample from 10 selected Egyptian universities by simple random sampling. The questionnaire was distributed via online platforms such as WhatsApp, Facebook, X, Telegram, and Messenger. Data about sociodemographic characteristics, students’ general knowledge about generative AI and its limitations, and their attitudes and practices, respectively, were collected.

**Results:**

About 2\3 (61.5%) of the studied students had satisfactory knowledge about generative AI and (44.7%) had a positive attitude. Males demonstrated significantly higher knowledge than females (69.3% vs. 55%, P-value = 0.003). Knowledge level also differs significantly by university (P-value < 0.001), with Suez-Canal and 6th October university students achieving the highest satisfactory scores. Key significant predictors that affect knowledge level include gender, university and study phase according to binary logistic regression results. Furthermore, there is a positive correlation between satisfactory knowledge and Practice level (*r* = 0.303, *P* < 0.001). Finally, most students use generative AI to check Grammar and prepare their homework or assignments.

**Conclusion and recommendations:**

There is moderate knowledge and positive attitude about AI that were reflected in medical students’ practice. However, knowledge about AI influenced by gender, university and study phase. Importantly, better knowledge significantly correlates with greater generative AI practice. Offering specialized courses or faculty modules about AI enhance awareness among Egyptian medical students. Such initiatives will enable them to gain knowledge and apply it effectively and wisely.

**Supplementary Information:**

The online version contains supplementary material available at 10.1186/s12909-025-07329-x.

## Background

Artificial intelligence (AI) is the development of computer systems to perform tasks requiring human intelligence, such as accurate visual perception, speech recognition, timely decision-making, and translation between different languages [1]. Generative artificial intelligence (GAI), a subset of AI, uses enormous amounts of data to generate new information in many formats, such as text, photos, video, audio, and code, making artificial intelligence more powerful and attractive than before [2].

AI is a revolutionary innovation in healthcare that has attracted the attention of clinicians, researchers, physicians, and medical device industry professionals [3]. Many fields have already integrated AI into clinical practice. At first, in education, where AI is beneficial in this process, AI can assess medical students in case-based e-learning [4] or history-taking by using virtual reality [5, 6]. The commonest use of AI in medicine has been in radiology; for example, it can detect several chest X-ray abnormalities with high accuracy [7]. AI applications have expanded to other specialties, such as detecting arrhythmia from ECG or distinguishing between innocent and pathological murmurs in paediatrics [8, 9], also ophthalmology, dermatology, and pathology have benefited from generative AI [10–16]. It is essential for medical students to understand this significant area and to be aware of its advantages and drawbacks to utilize it effectively.

This rapid rise of generative AI has prompted research into the knowledge, attitudes, and practices of healthcare students regarding AI technologies in general and generative AI specifically. Existing literature from different regions around the world discussed the students’ experiences. Such as the study conducted in Jordan showed moderate knowledge of AI, and different attitudes showing both skepticism about AI replacing human roles and an appreciation for its value, although practical integration into their studies was limited [17]. On the other hand, the study conducted in Egypt showed that nearly half of the dental students have basic knowledge and awareness of AI and its application in their field, also they support the use of AI in dental training, but they are afraid that AI will replace clinicians [18]. Also, in Saudi Arabia on nursing students revealed that personality traits affected their attitudes towards AI, with openness leading to positive attitudes. while neuroticism and agreeableness lead to negative attitudes [19]. Finally, according to a United States study, nursing students showed a cautiously optimistic attitude when they used generative AI, knowing that it can create familiar summaries, but the results from generative AI should be checked carefully [20].

Although these studies provide valuable insights into student perspectives toward AI and generative AI, there remains a need for a specific understanding of medical students’ knowledge, attitudes, and practices toward generative AI across multiple Egyptian universities. Although a study conducted at Ain Shams University provided initial insights for medical students from a single university [21]. A more comprehensive assessment is crucial. So, our study aims to assess Egyptian medical students’ knowledge, attitude, and practice about generative AI as a first step toward the possibility of incorporating this important tool into the Egyptian medical curriculum.

## Methodology

### Study design

This cross-sectional study involved medical students from 10 Egyptian medical faculties. Data collection occurred in March 2024.

### Sample size and sampling technique

The sample size was calculated to be 423 participant using open EPI program [22] with a 5% margin of error assuming that the percentage of medical students with knowledge of and attitudes toward generative AI was (57.7%) [21], and the number of medical students in the selected universities is 73,000, according to the Central Agency for Public Mobilization and Statistics (**CAPMAS)** 2022–2023 [23], with 10% non -response rate.

The medical students were enrolled as a convenience sample and were selected from 10 randomly selected Egyptian universities by a simple random sampling technique (10 out of 53 Egyptian universities). The enrolled universities were: Zagazig, Al-Azhar, Mansura, Minia, 6th October, Alexandria, Bani-Suef, Menoufia, Suez Canal, and Fayoum.

Students aged 18–25 years, of both sexes, and who had access to online platforms were included.

### Data collection

A semi-structured online questionnaire in English was designed via Google Forms. It was adapted from previous studies and modified to capture the necessary data on students’ knowledge, attitudes, and practices regarding generative AI [17, 24, 25]. The questionnaire was shared via online platforms such as WhatsApp, Facebook, X, Telegram, and Messenger.

The questionnaire was divided into 5 sections. The first one was for the consent obtained from the students. In the second one, we collected sociodemographic data about these students such as age, sex, university, study year, residency, current living situation, and studying phase (Academic phase: the first two years in the faculty and clinical phase: included the last three years). This division was based on the universities’ categorization in Egypt.

In the third part, we collected their general knowledge and their opinion about limitations of usage of generative AI. The fourth and fifth parts assessed their attitudes and practices, respectively.

### Scoring

The knowledge part about Generative AI consists of 8 questions. The total scores were calculated by awarding (1) for yes and (0) for no answers, making the maximum scoring level for each one (8 points) and the minimum (0 points). A cumulative score of (4 points) or more for each is considered a satisfactory knowledge level, and the students’ opinions about AI’s limitation part consists of 7 questions, with (1) score for yes answers and (0) for no answers.

Attitude and practice questions were 13 and 7, respectively. scoring was generated for attitude by awarding (1) for strongly disagree, (2) for disagree, (3) for neutral, (4) for agree, and (5) for strongly agree. Except for the second question “Suppose artificial intelligence makes a diagnosis, what would you prefer?” Scoring was made by awarding (1) for the generative AI misses almost no diagnosis but often gives a false alarm, (2) for the generative AI gives a false alarm about as often as it misses a diagnosis, and (3) for the generative AI rarely gives a false alarm but sometimes misses a diagnosis. This made the maximum score (63 points) and the minimum score (13 points) with people of (44 points) or more considered to have a good attitude toward generative AI, and a lower score considered a negative attitude toward generative AI.

Finally, the practice score was conducted by awarding (1) for Never, (2) for Rarely, (3) for often, (4) for most of the time, and (5) for all the time, so the score ranged from (35 to 7 points) with people of (17 points) or more score considered to have good practice of generative AI and lower score considered bad practice of generative AI.

### Questionnaire validation and piloting

A pilot study was conducted of 40 students, which was (10%) of the expected responses. Also, we assessed the data validation using Cronbach’s Alpha test to check the validity of the questions. Cronbach’s Alpha of (0.708) indicated good consistency all over the questionnaire.

### Statistical analysis

Statistical analysis was conducted via IBM SPSS version 27, Data were represented in tables and graphs, Continuous quantitative variables were expressed as mean ± SD & (range), and categorical qualitative variables were expressed as numbers and percentage, suitable statistical tests were used, after normality testing, Mann-Whitney and chi-square tests were conducted, Bonferroni correction for multiple tests was done. Pearson’s correlation coefficient (r) was calculated as a measure of the strength of the association between two numeric variables. Also, a binary logistic regression model was used to assess change in knowledge according to gender, the university, study phase, and residency. The results were considered highly statistically significant when the significance probability was less than (*P* < 0.001).

## Results

### Sociodemographic data

Among the 423 studied students, (54.6%) were female, and (45.6%) were male. The mean age was (21.65 ± 1.44) years old. A total of (78.3%) were from the clinical phase, and (21.7%) were from the academic phase. A majority of participants (91.1%) were from governmental universities, while (9.9%) were from private ones, and (66%) were from urban areas, as shown in Table 1.

**Table 1:** Sociodemographic characteristics of medical students participating in this study, Egypt 2024 (*n* = 423)

Characteristics of studied students		The studied students (423)	
		No.	%
Age (years)	Mean ± SD	21.65 ± 1.44	
Sex	Male	231	54.6%
	female	192	45.4%
University	Al-Azhar University	44	10.4%
	Alexandria University	72	17.0%
	Beni Suef University	39	9.2%
	Cairo University	41	9.7%
	Fayoum University	9	2.1%
	Mansoura University	61	14.4%
	Minia University	32	7.6%
	October 6 University	42	9.9%
	Suez Canal University	13	3.1%
	Zagazig University	70	16.5%
Academic Year	First-year	30	7.1%
	Second year	48	11.3%
	Fourth year	143	33.8%
	Third year	49	11.6%
	Fifth year	153	36.2%
Academic phase	Phase 1 (Academic phase)	92	21.7%
	Phase 2 (Clinical phase)	331	78.3%
Residence	Rural	144	34.0%
	Urban	279	66.0%
Current living situation	Living with others (family, friends, etc.)	381	90.1%
	Living alone	42	9.9%

We found that (45.4%) of the students had solid knowledge, and (63.8%) knew some application of generative AI in their field of interest; however, only (29.8%) knew what deep/machine learning was. Few students had courses to learn generative AI or were taught generative AI in medical curricula (23.9% and 29.6%), respectively. On the other hand, most students were familiar with generative AI in education and various generative AI tools and understood its barriers well (70.2%, 70.4%, and 69.5%), respectively.

Regarding AI usage limitation, (39%) of the studied students reported a lack of knowledge, while (34.8% and 32.9%) of them reported a lack of training centers and access/technical equipment problems, respectively, as shown in Tables 2 and 3.

**Table 2:** Distribution of knowledge satisfaction regarding generative AI among medical students participating in this study, Egypt 2024 (*n* = 423)

Knowledge about AI	Knowledge (No = 423)			
	Unsatisfactory		Satisfactory	
	No.	%	No.	%
Do you have a solid knowledge of the basics of AI?	231	54.6%	192	45.4%
Do you know what deep learning/machine learning is?	297	70.2%	126	29.8%
Do you know any application of AI in your field of interest?	153	36.2%	270	63.8%
Have you attended any previous online/offline courses regarding AI?	322	76.1%	101	23.9%
Have you ever been taught about AI in your undergraduate studies?	298	70.4%	125	29.6%
Does AI require a lot of labelled data to learn (data already processed by a human)?	156	36.9%	267	63.1%
Familiar with the concept of AI in education?	126	29.8%	297	70.2%
Familiar with the various AI tools available for educational purposes (Chat-GPT, Gemini, Bing, ……)?	125	29.6%	298	70.4%
Total knowledge score: Mean ± SD (Range)	1.82 ± 1.17 (0–3)		5.30 ± 1.21 (4–8)	

**Table 3:** Distribution of generative AI limitations among medical students participating in this study, Egypt 2024 (*n* = 423)

Limitations of AI usage	Studied students (*N* = 423)	
	No.	%
Lack of knowledge and expertise	**165**	**39.0%**
Lack of access/technical equipment	**139**	**32.9%**
Ethical and privacy concerns	**120**	**28.4%**
Lack of time due to educational burden	**112**	**26.5%**
Complexity of AI	**110**	**26.0%**
Limited integration in educational curricula	**102**	**24.1%**
Does AI require a lot of labelled data to learn (data already processed by a human)?	**147**	**34.8%**

**Fig. 1:**
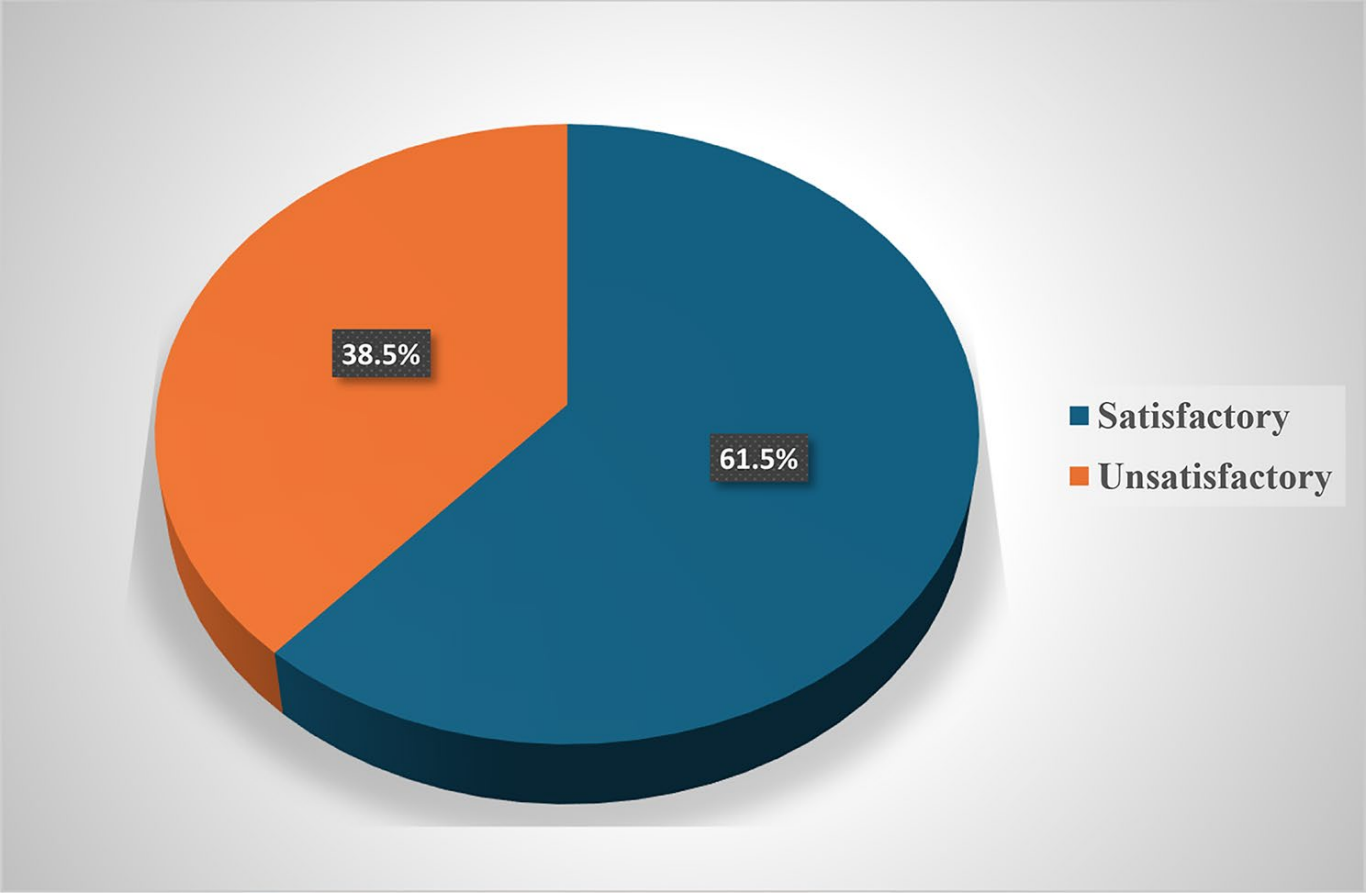
Knowledge satisfaction regarding generative AI among medical students participating in this Study, Egypt 2024 (*n* = 423). Pie diagram representing distribution of the patients studied according to satisfactory knowledge about AI

The total satisfactory level of general knowledge about generative AI was (61.5%), of which males had a statistically significant higher knowledge than females (69.3% versus 55%), respectively and (P-value = 0.003) (Fig. 1). Additionally, we found a statistically significant difference between universities (P-value < 0.001) as Suez-Canal and October 6th university students had the highest satisfactory scores (92.3% and 85.7%) respectively, and Mansoura and Beni Suef university students were the lowest (42.6% and 43.6%) respectively.

Additionally, the higher the academic year the higher the satisfactory scores as we found clinical phase students were higher than Academic one in satisfactory scores (74.4% and 58.6% respectively, P-value = 0.01). However, no statistically significant difference was found between urban and rural students (68.8% vs. 56.9% respectively, P-value = 0.17) (see supplementary material).

Finally, the level of general knowledge about generative AI had an impact on opinion about its limitations of usage as we found a statistically significance relationship between levels of knowledge and generative AI ethical and privacy concerns, lack of time due to education burden (P-value = 0.01) for both, The median of knowledge and limitation score was 4 and 2 out of a maximum possible score of 8 and 7 respectively.

### Students’ attitudes towards generative AI

**Fig. 2:**
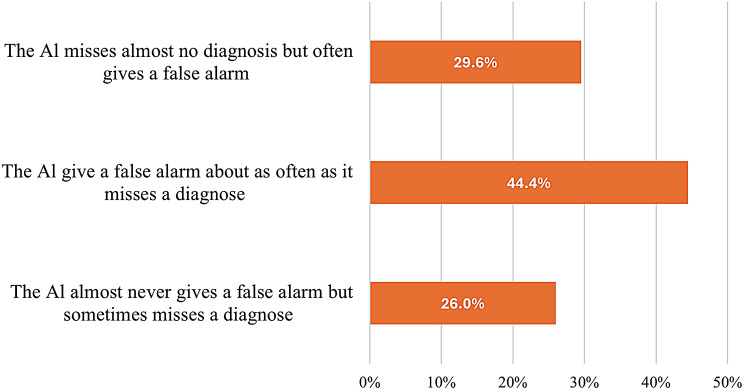
Preferences Regarding Generative AI Diagnosis Among Medical Students Participating in This Study, Egypt 2024 (*n* = 423). The graph shows the distribution of medical students’ preferences when artificial intelligence is used to make a medical diagnosis, highlighting the varying levels of trust and acceptance of AI-generated diagnostic insights

This study showed that (44.7%) of students had a positive attitude toward AI in the future, and approximately (75.1%) suggested improvement in the next 10 years. When AI makes a diagnosis, (44.4%) suggested that AI can give false alarms as often as it misses a diagnosis (Fig. 2).

**Fig. 3:**
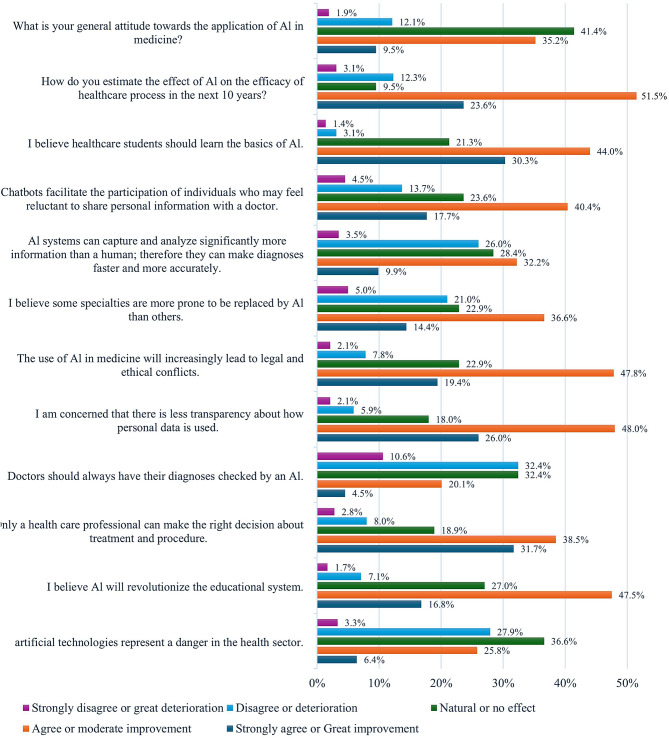
Attitude toward Generative AI Among Medical Students Participating in This Study, Egypt 2024 (*n* = 423). The graph illustrates the nuanced attitudes of medical students towards artificial intelligence, including perspectives on generative AI’s potential impact on medical diagnosis, treatment decisions, healthcare professionals’ roles, and others

In general, the most positive attitude among students toward generative AI was in the educational system: “(74.3%) agreed to want medical students to learn generative AI, (64.3%) saw that generative AI will revolutionize education”, and (58.1%) saw that generative AI can help patients who find it difficult to deal with doctors directly. Approximately (70%) agreed that healthcare professionals are only capable of making medical decisions. And (50%) saw that some specialties are prone to be replaced by generative AI, and (74%) were concerned with the transparency of the data used by generative AI; however, only (32.2%) considered generative AI dangerous to the healthcare sector (Fig. 3). (See Supplement material), The median attitude score was 44 out of a maximum possible score of 63.

### Students’ practice using generative AI tools

The highest percentage of the students often used generative AI for spelling and grammar checking (28.1%), preparing for homework or assignments (29.1%), conducting their research (30.7%), and idea generation and brainstorming (29.8%) in comparison to other responses.

However, the studied students rarely used generative AI to prepare for their exams (32.4%) and never used generative AI for personality development and other skills, such as courses (32.4%), while (43.3%) never used Al for personal choices or career guidance as shown in (Table 4).

**Table 4:** Distribution of practice regarding generative AI among medical students participating in this study, Egypt 2024 (*n* = 423)

Practice of AI	Practice (No = 423)				
	Never	Rarely	Often	Most of the time	All the time
	*N* (%)	*N* (%)	*N* (%)	*N* (%)	*N* (%)
How frequently do you use Al to prepare for your exams?	125 (29.6%)	137 (32.4%)	113 (26.7%)	35 (8.3%)	13 (3.1%)
How frequently do you use Al to prepare for your homework/assignment?	105 (24.8%)	116 (27.4%)	123 (29.1%)	59 (13.9%)	20 (4.7%)
How frequently do you use Al to conduct your research?	95 (22.5%)	90 (21.3%)	130 (30.7%)	76 (18%)	32 (7.6%)
How frequently do you use Al for idea generation and brainstorming?	107 (25.3%)	96 (22.7%)	126 (29.8%)	69 (16.3%)	25 (5.9%)
How frequently do you use Al for personal choices/career guidance?	183 (43.3%)	107 (25.3%)	80 (18.9%)	39 (9.2%)	14 (3.3%)
How frequently do you use Al for spelling and grammar checking?	105 (24.8%)	83 (19.6%)	119 (28.1%)	77 (18.2%)	39 (9.2%)
How frequently do you use AI for personality development and other skills, like courses?	137 (32.4%)	112 (26.5%)	90 (21.3%)	51 (12.1%)	33 (7.8%)

Concerning the generative AI tools used by the studied students, (37.1%) used Chat-GPT 3.5, about 1\3 of them (35.2%) used Chat-GPT 4, and fewer than (20%) used Microsoft Copilot, Grammarly, Gemini, Quill bot, and Perplexity AI; however, (12.5%) of the students used other generative AI tools, and (10.2%) of them did not use any generative AI tool. (see supplementary material). The median practice score was 17 (range 17–18) out of a maximum possible score of 35.

The current study found that there was statistically significant correlations between satisfactory knowledge and Practice level as the higher the knowledge the greater the practice using generative AI tools (coefficient *r* = 0.303, P-value < 0.001).

### Binary logistic regression

Logistic regression analysis shows that Male (OR = 1.871, CI (1.191, 2.941), P-value = 0.007), some universities like Al-Azhar University (P-value = 0.003), Beni Suef university (OR = 0.381, CI (0.150, 0.966), P-value = 0.042), October 6 university (OR = 3.551, CI (1. 135, 11.111), P-value = 0.029), and clinical phase (OR = 0.539, CI (0.296, 0.981), P-value = 0.043) were statistically significant independent predictors of knowledge about generative AI. A logistic regression was performed to ascertain the effects of sex, university, Academic or clinical phase, residency, and living situation on the participants of the study in relation to knowledge. The logistic regression model was highly statistically significant, (χ^2^ = 47.209, P-value < 0.001). The model explained (Nagelkerke *R*^*2*^ = 14.3%) of the variance in knowledge changes and correctly classified (67%) of cases. Male participants were (1.87 times) more likely to have more knowledge about generative AI than females and Beni Suef was (0.38 times) more likely to have less knowledge about generative AI than Al-Azhar University.

While October 6th students were (3.5 times) more likely to have more knowledge than Al-Azhar University students. Finally, the clinical phase was (0.54 times) more likely to have less knowledge than the academic phase (see supplementary material).

## Discussion

Artificial intelligence has gained considerable importance in the medical field in recent years [26, 27]Therefore, it is important to assess the knowledge, attitudes, and practices of healthcare professionals in different specialties, especially the coming generation of medical students, regarding generative AI in Egypt.

### Knowledge about AI

Our study revealed a moderate level of knowledge among medical students about understanding generative AI regarding its meaning, usage, application in the medical field, and its limitations and ethical concerns.

Our findings are similar to those of several cross-sectional studies in several countries. Baigi, 2023 [28] was the first systematic review to assess the knowledge, attitude, and skills among medical, dental, and pharmacist students. It summarized 38 cross-sectional studies conducted from 2019 to 2022 around the world. This study was conducted when generative AI was first introduced to the public. They revealed that most students had limited knowledge about generative AI with a positive attitude.

This could be explained as generative AI was a new trend with little understanding of its meaning, lack of access to free paid apps or internet, and lack of teaching centers for new trends about AI, also it could be expected with the medical field studying overwhelming.

Similar findings were presented in other studies among medical students in different Arabic countries such as Saudi Arabia [29] and Kuwait [30].and the study which was conducted by Al-Qerem, 2023 [17] among Jordan medical, dental, and pharmacy students, Al-Qerem, 2023 stated that there was a moderate level of knowledge with a positive attitude and little practice. They interestingly found a positive association between the type of college and both knowledge and attitude, as medical students had more knowledge and attitude scores than dental and pharmacy students.

This could be explained as medical students might be more prone to use generative AI tools in their future work such as in surgical specialties or radiology with much interest in learning AI.

In the current study, there was a statistically significant difference between males and females in knowledge scores where males had a higher knowledge than females (69.3% versus 55%) respectively, in the contrary to the study conducted by Elchaghaby, 2025 [18] in Egypt to assess the knowledge, attitude, and perception of dental students.

This difference in knowledge and usage of AI tools between men and women could be attributed to that men generally have higher engagement and interest in AI, while some women may find interacting with AI tools less appealing due to perceived utility and the specific way of thinking required.

In the current study, a positive association was found between the university and knowledge scores, Students from the 6th October University, which is a private university, had the highest knowledge score followed by Al Azhar University, this can be explained as 6th October students had access to more technology in their educational process than other governmental universities.

Additionally, the Clinical phase students had more knowledge than the academic phase. This could be explained as the clinical phase students learn about internal medicine, surgery, and other specialties making it easier for them to understand the impact of generative AI on the medical field.

### Attitude towards AI

In the current study, despite moderate knowledge, most of the studied medical students had a positive attitude towards generative AI, as they consider it an effective tool for medical education and helping patients communicate with doctors. While they still had some concerns about replacing some medical specialties. Most students (67.2%) had some concerns about ethical considerations of using generative AI.

These findings are similar to the study conducted by Elchaghaby, 2025 [18] to assess the knowledge, attitude, and perception of dental students from Cario and Russian dental schools in Egypt, they found a moderate knowledge level with a positive attitude toward generative AI.

Khater, 2023 [21] conducted a study on medical students at Ain Shams University to assess the knowledge and attitude of medical students in Egypt. Khater, stated the same findings where (41.2%) had good knowledge and (57.7%) had a positive attitude toward generative AI.

Hasan, 2024 [31] conducted a cross-sectional study on pharmacy students from different countries around the world including Egypt and also reported that there was moderate knowledge with a positive attitude towards generative AI.

### Practice of generative AI

In the current study, moderate knowledge and positive attitude were reflected in medical students’ practice, as most of them use generative AI tools in grammar checking, doing tasks, and homework, and conducting research.

In the contrary, in another cross-sectional study in two different medical schools in Germany, students were asked about AI literacy knowledge and attitudes, which revealed slightly different findings than the current study, as the findings revealed that students had positive attitudes, the ability to critically appraise AI and their use in practice was high; however, their technical understanding was limited [32].

This could be explained as German students and developed countries use technology in their education more frequently than those in the Arab world do and being exposed to incorporation of generative AI learning in medical their curricula.

For generative AI’s increasing importance, several trials have been conducted to incorporate generative AI learning in medical curricula. For example, Moldt, 2023 [25] was an elective course for 12 medical students in Germany, that tried to increase medical awareness. The course succeeds in increasing students’ awareness and attitude paving the way for German medical schools to teach generative AI in their curricula.

Another course was conducted on nurse students in Portland. Hawk, 2024 [20] assessed the influence of applying generative AI in research compared with ordinary research with guidance on the way it is used. They reported positive outcomes as students found it easy to use generative AI tools and find the right information easily.

Finally, male sex, private universities and clinical phase were statistically significant independent predictors of knowledge about generative AI. This study also highlights the need to incorporate AI knowledge into medical curricula.

### Study limitations

Our study has several limitations. Our sample was a convenient sample, which can lead to potential selection bias, potential recall bias, and responder bias as the questionnaire was self-reported online. The Nagelkerke R² value (14.3%) indicates modest predictive power of satisfactory knowledge. The size of our sample is too small to extend our conclusions to all medical universities in Egypt. Another potential limitation of our study is the length of the questionnaire, which may discourage some students from completing it. Additionally, since the questionnaire was administered online, it led to exclusion of students with limited digital access. Finally, as the questionnaire is about a new field, “Generative AI,” most of the students are already interested in this field, which results in selection bias. To solve these problems, several studies should be performed in medical schools in Egypt with larger sample sizes and using diverse sample methods for better interpretation.

Although that several strengths exist as the current study assessed the knowledge, attitudes, and practices of generative AI in several medical schools in Egypt not only one medical school discussing this important field among a wide range of medical students in different Egyptian universities, providing a view of Egyptian medical students’ thinking and interpretation of generative AI.

## Conclusion

Our study explored the knowledge, attitudes, and practices of Egyptian medical students towards generative AI and revealed that it is crucial for students to learn the basics of generative AI and to use it in their daily practice, as they will use it in their professional work in the future. It also sheds light on the importance of medical schools in Egypt incorporating teaching generative AI and generative AI tools into their medical curriculum to prepare their students for future challenges.

We recommend conducting specific modules to teach medical students the meaning of generative AI, its usage, and its ethical considerations. Also to help them incorporate generative AI tools into their learning process such as helping with single-based answer questions, finally faculties should provide technical support to students wishing to learn AI and guide them to solve any problems regarding the usage of these tools.

## Electronic supplementary material

Below is the link to the electronic supplementary material.

Supplementary Material 1

Supplementary Material 2

## Data Availability

The dataset was collected from the answer of participants to our questionnaire and here is the google sheet of these answers results: https://docs.google.com/spreadsheets/d/1r025zczOALKjjBXk8ZsgIe7tla9jSSm1/edit?usp=sharing_ouid=110869071363678691856_rtpof=true_sd=true.

## References

[1] Mousavi Baigi SF, Sarbaz M, Ghaddaripouri K, Ghaddaripouri M, Mousavi AS, Kimiafar K. Attitudes, knowledge, and skills towards artificial intelligence among healthcare students: A systematic review. Health Sci Rep. 2023;6. 10.1002/hsr2.1138 PMC10009305 36923372

[2] Shoja MM, Van de Ridder JMM, Rajput V. The emerging role of generative artificial intelligence in medical education, research, and practice. Cureus. 2023. 10.7759/cureus.40883. 10.7759/cureus.40883 PMC10363933 37492829

[3] Hasan Sapci A, Aylin Sapci H. Artificial intelligence education and tools for medical and health informatics students: systematic review. JMIR Med Educ. 2020;6. 10.2196/19285 PMC7367541 32602844

[4] Khumrin P, Ryan A, Judd T, Verspoor K. Diagnostic machine learning models for acute abdominal pain: towards an e-Learning tool for medical students. Stud Health Technol Inf. 2017;245:447–51. 29295134

[5] Randhawa GK, Jackson M. The role of artificial intelligence in learning and professional development for healthcare professionals. Healthc Manage Forum. 2020;33:19–24. 31802725 10.1177/0840470419869032

[6] Maicher KR, Zimmerman L, Wilcox B, Liston B, Cronau H, Macerollo A, et al. Using virtual standardized patients to accurately assess information gathering skills in medical students. Med Teach. 2019;41:1053–9. 31230496 10.1080/0142159X.2019.1616683

[7] Rajpurkar P, Irvin J, Ball RL, Zhu K, Yang B, Mehta H et al. Deep learning for chest radiograph diagnosis: A retrospective comparison of the CheXNeXt algorithm to practicing radiologists. PLoS Med. 2018;15. 10.1371/journal.pmed.1002686 PMC6245676 30457988

[8] Hannun AY, Rajpurkar P, Haghpanahi M, Tison GH, Bourn C, Turakhia MP, et al. Cardiologist-level arrhythmia detection and classification in ambulatory electrocardiograms using a deep neural network. Nat Med. 2019;25:65–9. 30617320 10.1038/s41591-018-0268-3 PMC6784839

[9] Zhou G, Chien C, Chen J, Luan L, Chen Y, Carroll S, et al. Identifying pediatric heart murmurs and distinguishing innocent from pathologic using deep learning. Artif Intell Med. 2024;153:102867. 38723434 10.1016/j.artmed.2024.102867

[10] Honavar SG. Artificial intelligence in ophthalmology - Machines think! Indian J Ophthalmol. 2022;70:1075. 35325987 10.4103/ijo.IJO_644_22 PMC9240552

[11] Shahriari MH, Sabbaghi H, Asadi F, Hosseini A, Khorrami Z. Artificial intelligence in screening, diagnosis, and classification of diabetic macular edema: A systematic review. Surv Ophthalmol. 2023;68:42–53. 35970233 10.1016/j.survophthal.2022.08.004

[12] Topol EJ. High-performance medicine: the convergence of human and artificial intelligence. Nat Med. 2019;25:44–56. 30617339 10.1038/s41591-018-0300-7

[13] Hainc N, Federau C, Stieltjes B, Blatow M, Bink A, Stippich C. The bright, artificial intelligence-augmented future of neuroimaging reading. Front Neurol. 2017;8:SEP. 10.3389/fneur.2017.00489 PMC5613097 28983278

[14] Wang D, Khosla A, Gargeya R, Irshad H, Beck AH. Deep Learning for Identifying Metastatic Breast Cancer. 2016.

[15] Kelly M, Ellaway R, Scherpbier A, King N, Dornan T. Body pedagogics: embodied learning for the health professions. Med Educ. 2019;53:967–77. 31216603 10.1111/medu.13916

[16] Esteva A, Kuprel B, Novoa RA, Ko J, Swetter SM, Blau HM, et al. Dermatologist-level classification of skin cancer with deep neural networks. Nature. 2017;542:115–8. 28117445 10.1038/nature21056 PMC8382232

[17] Al-Qerem W, Eberhardt J, Jarab A, Al Bawab AQ, Hammad A, Alasmari F, et al. Exploring knowledge, attitudes, and practices towards artificial intelligence among health professions’ students in Jordan. BMC Med Inf Decis Mak. 2023;23:288. 10.1186/s12911-023-02403-0 PMC10722664 38098095

[18] Elchaghaby M, Wahby R. Knowledge, attitudes, and perceptions of a group of Egyptian dental students toward artificial intelligence: a cross-sectional study. BMC Oral Health. 2025;25:1–7. 39754101 10.1186/s12903-024-05282-7 PMC11697735

[19] Salem GMM, El-Gazar HE, Mahdy AY, Alharbi TAF, Zoromba MA. Nursing Students’ Personality Traits and Their Attitude toward Artificial Intelligence: A Multicenter Cross-Sectional Study. J Nurs Manag. 2024;2024. 10.1155/2024/6992824 PMC11919015 40224799

[20] Hawk H, Coriasco M, Jones JR. Generative Artificial Intelligence. Nurse Educ. 2024. 10.1097/NNE.0000000000001736 10.1097/NNE.0000000000001736 39288334

[21] Khater AS, Zaaqoq AA, Wahdan MM, Ashry S. Knowledge and attitude of Ain Shams university medical students towards artificial intelligence and its application in medical education and practice. Educational Res Innov J. 2023;3:29–42.

[22] Epi Info™ | CDC. https://www.cdc.gov/epiinfo/index.html. Accessed 13 Jan 2025.

[23] Capmas. https://www.capmas.gov.eg/HomePage.aspx. Accessed 31 Jan 2025.

[24] Busch F, Hoffmann L, Truhn D, Palaian S, Alomar M, Shpati K, et al. International pharmacy students’ perceptions towards artificial intelligence in medicine—A multinational, multicentre cross-sectional study. Br J Clin Pharmacol. 2024;90:649–61. 37728146 10.1111/bcp.15911

[25] Moldt J-A, Festl-Wietek T, Madany Mamlouk A, Nieselt K, Fuhl W, Herrmann-Werner A. Chatbots for future docs: exploring medical students’ attitudes and knowledge towards artificial intelligence and medical chatbots. Med Educ Online. 2023;28:2182659. 36855245 10.1080/10872981.2023.2182659 PMC9979998

[26] Carobene A, Cabitza F, Bernardini S, Gopalan R, Lennerz JK, Weir C, et al. Where is laboratory medicine headed in the next decade? Partnership model for efficient integration and adoption of artificial intelligence into medical laboratories. Clin Chem Lab Med. 2023;61:535–43. 36327445 10.1515/cclm-2022-1030

[27] Davenport T, Kalakota R. The potential for artificial intelligence in healthcare. Future Healthc J. 2019;6:94. 10.7861/futurehosp.6-2-94 PMC6616181 31363513

[28] Mousavi Baigi SF, Sarbaz M, Ghaddaripouri K, Ghaddaripouri M, Mousavi AS, Kimiafar K. Attitudes, knowledge, and skills towards artificial intelligence among healthcare students: A systematic review. Health Sci Rep. 2023;6:e1138. 36923372 10.1002/hsr2.1138 PMC10009305

[29] Alghamdi SA, Alashban Y. Medical science students’ attitudes and perceptions of artificial intelligence in healthcare: A National study conducted in Saudi Arabia. J Radiat Res Appl Sci. 2024;17:100815.

[30] Buabbas AJ, Miskin B, Alnaqi AA, Ayed AK, Shehab AA, Syed-Abdul S et al. Investigating students’ perceptions towards artificial intelligence in medical education. Healthc (Basel). 2023;11. 10.3390/healthcare11091298 PMC10178742 37174840

[31] Hasan HE, Jaber D, Tabbah S, Al, Lawand N, Habib HA, Farahat NM. Knowledge, attitude and practice among pharmacy students and faculty members towards artificial intelligence in pharmacy practice: A multinational cross-sectional study. PLoS ONE. 2024;19. 10.1371/journal.pone.0296884 PMC10906880 38427639

[32] Laupichler MC, Aster A, Meyerheim M, Raupach T, Mergen M. Medical students’ AI literacy and attitudes towards AI: a cross-sectional two-center study using pre-validated assessment instruments. BMC Med Educ. 2024;24:401. 38600457 10.1186/s12909-024-05400-7 PMC11007897

