# Supplementary material for: Medical students’ knowledge, attitudes, and practices toward generative artificial intelligence in Egypt 2024: a Cross-Sectional study
Source: BMC Med Educ. 2025 May 28;25:790. doi: 10.1186/s12909-025-07329-x (PMC12117742; doi:10.1186/s12909-025-07329-x)
Supplement: Supplementary file 1 — Supplementary Material 1 [file 12909_2025_7329_MOESM1_ESM.pdf]

Knowledge and Attitude Towards Generative AI Among University Medical Students in Egypt

Artificial intelligence has greatly evolved with the advent of Generative AI, surpassing the capabilities of previous AI systems. In the past, AI performed specific tasks and could not do much beyond what it was programmed to do. How peopleever, with the introduction of Generative AI tools like ChatGPT-4, Bing, or Gemini, AI can now create its own content, making it much closer to human-like performance. This new technology brings both advantages and disadvantages to various sectors of our lives, making it crucial to understand it thoroughly to reap its benefits. In the medical field, AI has multiple applications, such as in radiology, and with the emergence of Generative AI, it is vital for us to comprehend its strengths and weaknesses. Therefore, our study aims to assess the level of knowledge and attitude about Generative AI among medical students in Egypt.

* Indicates required question

1. By proceeding, you consent to share your responses with the researchers. *

Mark only one oval.

- I agree

2. Code * Dropdown

Mark only one oval.

- 0
- 1
- 2
- 3
- 4
- 5
- 6
- 7

Sociodemographic

3. How old are you? *

Dropdown

Mark only one oval.

- 18
- 19
- 20
- 21
- 22
- 23
- 24
- 25
-

4. What is your gender? *

Mark only one oval.

- Male
- Female

5. What is your university? *

Dropdown

Mark only one oval.

- Cairo University
- Al-Azhar University
- Alexandria University
- Zagazig University
- Minia University
- Beni Suef University
- Fayoum University
- Mansoura University
- Suez Canal University
- October 6 University

6. What is your academic year? *

Mark only one oval.

- First year
- Second year
- Third year
- Fourth year
- Fifth year

7. Which phase are you in? *

Mark only one oval.

- Phase 1 (Academic phase
- Phase 2 (clinical phase)

8. What is your residency? *

Mark only one oval.

- Urban
- Rural

9. What is your current living situation? *

Mark only one oval.

- Living alone
- Living with other (family, friends, etc)

Knowledge

10. Based on your Knowledge of artificial intelligence, answer the following questions *

Mark only one oval per row.

	Yes	No
Do you have a solid knowledge of the basics of AI?	⬭	⬭
Do you know what deep learning/machine learning is?	⬭	⬭
Do you know any application of AI in your field of interest	⬭	⬭
Have you attended any previous online/offline courses regarding AI?	⬭	⬭
Have you ever been taught about AI in your under-graduate studies?	⬭	⬭
Does AI require a lot of labeled data to learn (data already processed by a human)?	⬭	⬭
Familiar with the concept of AI in education ?	⬭	⬭
Familiar with the various AI tools available for educational purposes(Chat-gpt, Gemini, Bing, ...)?	⬭	⬭

11. I understand the barriers of applying AI in medicine *

Mark only one oval.

- Yes
- No

12. If you answered yes, what are the barriers do you face?

Check all that apply.

- [ ] Lack of knowledge and expertise
- [ ] Lack of access/technical equipment
- [ ] Ethical and privacy concerns
- [ ] Lack of time due to educational burden
- [ ] Complexity of AI
- [ ] Limited integration in educational curricula
- [ ] Lack of teaching centers and hands-on applications
- [ ] Other: ______________________

Attitude

13. What is your general attitude towards the application of AI in medicine? *

Mark only one oval.

- Strong negative
- Negative
- Natural
- Positive
- Strongly positive

14. How do you estimate the effect of AI on the efficacy of healthcare process in the next 10 years? *

Mark only one oval.

- Great deterioration
- moderate Deterioration
- No effect
- moderate Improvement
- Great improvement

15. Suppose artificial intelligence makes a diagnosis, what would you prefer? *

Mark only one oval.

- The AI misses almost no diagnosis but often gives a false alarm,
- The AI give a false alarm about as often as it misses a diagnose,
- The AI almost never gives a false alarm but sometimes misses a diagnose)

16. I believe healthcare students should learn the basics of AI *

Mark only one oval.

- strongly agree
- agree
- natural
- disagree
- strongly disagree

17. Chatbots facilitate the participation of individuals who may feel reluctant to share * personal information with a doctor
تسهل الدردشة الآلية مشاركة الأفراد الذين قد يترددون في مشاركة المعلومات الشخصية مع الطبيب

Mark only one oval.

- strongly agree
- agree
- natural
- disagree
- strongly disagree

18. AI systems can capture and analyze significantly more information than a human; therefore they can make diagnoses faster and more accurately. *

Mark only one oval.

- strongly agree
- agree
- natural
- disagree
- strongly disagree

19. I believe some specialties are more prone to be replaced by AI than others, *

Mark only one oval.

- strongly agree
- agree
- natural
- disagree
- strongly disagree

20. The use of AI in medicine will increasingly lead to legal and ethical conflicts *

Mark only one oval.

- strongly agree
- agree
- natural
- disagree
- strongly disagree

21. I am concerned that there is less transparency about how personal data is used. *
أنا قلق من أن يكون هناك نقص في الشفافية حول استخدام البيانات الشخصية

Mark only one oval.

- strongly agree
- agree
- natural
- disagree
- strongly disagree

22. طبDoctors should always have their diagnoses checked by an AI *

Mark only one oval.

- strongly agree
- Agree
- Neutral
- Disagree
- Strongly Disagree

23. Only a health care professional can make the right decision about treatment and * procedure.

Mark only one oval.

- strongly agree
- agree
- natural
- disagree
- strongly disagree

24. I believe AI will revolutionize the educational system *

Mark only one oval.

- strongly agree
- agree
- natural
- disagree
- strongly disagree

25. artificial technologies represent a danger in the health sector? *

Mark only one oval.

- strongly agree
- agree
- natural
- disagree
- strongly disagree

26. In your opinion, does artificial technologies represent an advantage in medical field, and why? *

Practice

27. Which Generative AI tools do you usually use? *

Check all that apply.

- [ ] Chat-GPT3.5
- [ ] Chat-GPT4
- [ ] Microsoft Copilot
- [ ] Gemini
- [ ] Perplexity AI
- [ ] Grammarly
- [ ] QuillBot
- [ ] Magic school ai
- [ ] Cohere
- [ ] Claude
- [ ] Poe
- [ ] Other: ____________________

28. How frequently do you use AI to prepare for your exams? *

Mark only one oval.

- ( ) All the time
- ( ) most of the time
- ( ) often
- ( ) Rarely
- ( ) Never

29. How frequently do you use AI to prepare for your homework/assignment? *

Mark only one oval.

- ( ) All the time
- ( ) most of the time
- ( ) often
- ( ) Rarely
- ( ) Never

30. How frequently do you use AI to conduct your research? *

Mark only one oval.

- All the time
- most of the time
- often
- Rarely
- Never

31. How frequently do you use AI for idea generation and brainstorming? *

Mark only one oval.

- All the time
- most of the time
- often
- Rarely
- Never

32. How frequently do you use AI for personal choices/career guidance? *

Mark only one oval.

- All the time
- most of the time
- often
- Rarely
- Never

33. How frequently do you use AI for spelling and grammar checking? *

Mark only one oval.

- All the time
- most of the time
- often
- Rarely
- Never

34. How frequently do you use AI for personality development and other skills, like courses? *

Mark only one oval.

- All the time
- most of the time
- often
- Rarely
- Never
