# Supplementary material for: Medical students’ knowledge, attitudes, and practices toward generative artificial intelligence in Egypt 2024: a Cross-Sectional study
Source: BMC Med Educ. 2025 May 28;25:790. doi: 10.1186/s12909-025-07329-x (PMC12117742; doi:10.1186/s12909-025-07329-x)
Supplement: Supplementary file 2 — Supplementary Material 2 [file 12909_2025_7329_MOESM2_ESM.docx]

**Table (1):** **Sociodemographic characteristic data of the participants studied (N= 423)**

| **Characteristics of studied participants** | | **The studied participants (423)** | |
| --- | --- | --- | --- |
|  |  | **No.** | **%** |
| **Age (years)** | **Mean ± SD** | 21.65 ± 1.44 | |
| **Sex** | **Male** | 231 | 54.6% |
|  | **female** | 192 | 45.4% |
| **University** | **Al-Azhar University** | 44 | 10.4% |
|  | **Alexandria University** | 72 | 17.0% |
|  | **Beni Suef University** | 39 | 9.2% |
|  | **Cairo University** | 41 | 9.7% |
|  | **Fayoum University** | 9 | 2.1% |
|  | **Mansoura University** | 61 | 14.4% |
|  | **Minia University** | 32 | 7.6% |
|  | **October 6 University** | 42 | 9.9% |
|  | **Suez Canal University** | 13 | 3.1% |
|  | **Zagazig University** | 70 | 16.5% |
| **Academic Year** | **First year** | 30 | 7.1% |
|  | **Second year** | 48 | 11.3% |
|  | **Fourth year** | 143 | 33.8% |
|  | **Third year** | 49 | 11.6% |
|  | **Fifth year** | 153 | 36.2% |
| **Academic phase** | **Phase 1 (Academic phase)** | 92 | 21.7% |
|  | **Phase 2 (Clinical phase)** | 331 | 78.3% |
| **Residence** | **Rural** | 144 | 34.0% |
|  | **Urban** | 279 | 66.0% |
| **Current living situation** | **Living with others (family, friends, etc.)** | 381 | 90.1% |
|  | **Living alone** | 42 | 9.9% |

**Table (2):** **Distribution of students' total knowledge about AI (No=423).**

| **Knowledge about AI** | **Knowledge (No=423)** | | | |
| --- | --- | --- | --- | --- |
|  | **Un satisfactory**  **No** | | **Satisfactory**  **Yes** | |
|  | **No** | **%** | **No** | **%** |
| Do you have a solid knowledge of the basics of AI? | 231 | 54.6% | 192 | 45.4% |
| Do you know what deep learning/machine learning is? | 297 | 70.2% | 126 | 29.8% |
| Do you know any application of AI in your field of interest? | 153 | 36.2% | 270 | 63.8% |
| Have you attended any previous online/offline courses regarding AI? | 322 | 76.1% | 101 | 23.9% |
| Have you ever been taught about AI in your undergraduate studies? | 298 | 70.4% | 125 | 29.6% |
| Does AI require a lot of labelled data to learn (data already processed by a human)? | 156 | 36.9% | 267 | 63.1% |
| Familiar with the concept of AI in education? | 126 | 29.8% | 297 | 70.2% |
| Familiar with the various AI tools available for educational purposes (Chat-gpt, Gemini, Bing, ……)? | 125 | 29.6% | 298 | 70.4% |
| Total knowledge score Mean ± SD  (Range) | 1.82 ± 1.17  (0 - 3) | | 5.30 ± 1.21  (4 – 8) | |

**Figure (1):** **Pie diagram representing distribution of the patients studied according to satisfactory knowledge about AI (No =423).**

**Table (3):** **Relation between the socio-economic characteristics of the studied participants and their level of knowledge.**

| **Characteristics of studied participants** | | **Knowledge** | | | | **p- value** |
| --- | --- | --- | --- | --- | --- | --- |
|  |  | **Unsatisfactory**  **< 50%** | | **satisfactory**  **≥ 50%** | |  |
|  |  | **No=**  **(163)** | **%** | **No= (260)** | **%** |  |
| **Age (years)** | **Mean ± SD** | 21.85 ± 1.32 | | 21.38 ± 1.49 | | **0.019*** |
| **Sex** | **Male** | 59 | 30.7% | 133 | 69.3% | **0.003*** |
|  | **female** | 104 | 45.0% | 127 | 55.0% |  |
| **University** | **Al-Azhar University** | 16 | 36.4% | 28 | 63.6% | **<0.001*** |
|  | **Alexandria University** | 29 | 40.3% | 43 | 59.7% |  |
|  | **Beni Suef University** | 22 | 56.4% | 17 | 43.6% |  |
|  | **Cairo University** | 13 | 31.7% | 28 | 68.3% |  |
|  | **Fayoum University** | 4 | 44.4% | 5 | 55.6% |  |
|  | **Mansoura University** | 35 | 57.4% | 26 | 42.6% |  |
|  | **Minia University** | 13 | 40.6% | 19 | 59.4% |  |
|  | **October 6 University** | 6 | 14.3% | 36 | 85.7% |  |
|  | **Suez Canal University** | 1 | 7.7% | 12 | 92.3% |  |
|  | **Zagazig University** | 24 | 34.3% | 46 | 65.7% |  |
| **Academic phase** | **Academic Phase** | 20 | 25.6% | 58 | 74.4% | **0.010*** |
|  | **Clinical Phase** | 143 | 41.4% | 202 | 58.6% |  |
| **Residence** | **Rural** | 62 | 43.1% | 82 | 56.9% | 0.170 |
|  | **Urban** | 101 | 36.2% | 178 | 63.8% |  |

***#Chi- square test • Mann- Whitney U test***

***P-value <0.05***

****Statistical significance***

**Table (4):** **Relation between limitation of AI usage and knowledge about AI (No=423)**

| **Variable** | **Knowledge** | | | | **p- value** |
| --- | --- | --- | --- | --- | --- |
|  | **Unsatisfactory**  **< 50%** | | **satisfactory**  **≥ 50%** | |  |
|  | **No=**  **(163)** | **%** | **No= (260)** | **%** |  |
| **Lack of knowledge and expertise** | 61 | 37.4% | 104 | 40% | 0.597 |
| **Lack of access/technical equipment** | 54 | 33.1% | 85 | 32.7% | 0.926 |
| **Ethical and private concerns** | 35 | 21.5% | 85 | 32.7% | **0.013*** |
| **Lack of time due to educational burden** | 32 | 19.6% | 80 | 30.8% | **0.012*** |
| **Complexity of AI** | 43 | 26.4% | 67 | 25.8% | 0.889 |
| **Limited integration in educational curriculum** | 35 | 21.5% | 67 | 25.8% | 0.315 |
| **Does AI require a lot of labelled data to learn (data already processed by a human)?** | 51 | 31.3% | 96 | 36.9% | 0.236 |

***#Chi- square test***

***P-value <0.05***

****Statistical significance***

**Figure (2): Bar graph representing distribution of satisfactory knowledge among different universities included in the study.**

**Table (5):** **Distribution of students' total Attitude towards AI (No=423).**

| **Attitude towards AI** | **Attitude (No=423)** | | | | |
| --- | --- | --- | --- | --- | --- |
|  | **Strongly agree** | **Agree** | **Natural** | **Disagree** | **Strongly disagree** |
|  | **N (%)** | **N (%)** | **N (%)** | **N (%)** | **N (%)** |
| **What is your general attitude towards the application of Al in medicine?** | 10  (9.5%) | 149  (35.2%) | 175  (41.4%) | 51  (12.1%) | 8  (1.9%) |
| **How do you estimate the effect of Al on the efficacy of healthcare process in the next 10 years?** | 100  (23.6%) | 218  (51.5%) | 40  (9.5%) | 52  (12.3%) | 13  (3.1) |
| **I believe healthcare students should learn the basics of Al** | 128  (30.3%) | 186  (44.0%) | 90  (21.3%) | 13  (3.1%) | 6  (1.4%) |
| **Chatbots facilitate the participation of individuals who may feel reluctant to share personal information with a doctor** | 75  (17.7%) | 171  (40.4%) | 100  (23.6%) | 58  (13.7%) | 19  (4.5%) |
| **Al systems can capture and analyze significantly more information than a human; therefore, they can make diagnoses faster and more accurately.** | 42  (9.9%) | 136  (32.2%) | 120  (28.4%) | 110  (26.0%) | 15  (3.5%) |
| **I believe some specialties are more prone to be replaced by Al than others,** | 61  (14.4%) | 155  (36.6) | 97  (22.9%) | 89  (21.0%) | 21  (5.0%) |
| **The use of Al in medicine will increasingly lead to legal and ethical conflicts** | 82  (19.4%) | 202  (47.8%) | 97  (22.9%) | 33  (7.8%) | 9  (2.1%) |
| **I am concerned that there is less transparency about how personal data is used.** | 110  (26.0%) | 203  (48.0%) | 76  (18.0%) | 25  (5.9%) | 9  (2.1%) |
| **Doctors should always have their diagnoses checked by an Al** | 19  (4.5%) | 85  (20.1%) | 137  (32.4%) | 137  (32.4%) | 45  (10.6%) |
| **Only a health care professional can make the right decision about treatment and procedure.** | 134  (31.7%) | 163  (38.5%) | 80  (18.9%) | 34  (8.0%) | 12  (2.8%) |
| **I believe Al will revolutionize the educational system** | 71  (16.8%) | 201  (47.5%) | 114  (27.0%) | 30  (7.1%) | 7  (1.7%) |
| **artificial technologies represent a danger in the health sector?** | 27  (6.4%) | 109  (25.8%) | 155  (36.6%) | 118  (27.9%) | 14  (3.3%) |

| **Practice of AI** | **Practice (No=423)** | | | | |
| --- | --- | --- | --- | --- | --- |
|  | **Never** | **Rarely** | **Often** | **Most of the time** | **All the time** |
|  | **N (%)** | **N (%)** | **N (%)** | **N (%)** | **N (%)** |
| **How frequently do you use Al to prepare for your exams?** | 125  (29.6%) | 137  (32.4%) | 113  (26.7%) | 35  (8.3%) | 13  (3.1%) |
| **How frequently do you use Al to prepare for your homework/assignment?** | 105  (24.8%) | 116  (27.4%) | 123  (29.1%) | 59  (13.9%) | 20  (4.7%) |
| **How frequently do you use Al to conduct your research?** | 95  (22.5%) | 90  (21.3%) | 130  (30.7%) | 76  (18%) | 32  (7.6%) |
| **How frequently do you use Al for idea generation and brainstorming?** | 107  (25.3%) | 96  (22.7%) | 126  (29.8%) | 69  (16.3%) | 25  (5.9%) |
| **How frequently do you use Al for personal choices/career guidance?** | 183  (43.3%) | 107  (25.3%) | 80  (18.9%) | 39  (9.2%) | 14  (3.3%) |
| **How frequently do you use Al for spelling and grammar checking?** | 105  )24.8%( | 83  )19.6%( | 119  )28.1%( | 77  )18.2%( | 39  )9.2%( |
| **How frequently do you use AI for personality development and other skills, like courses?** | 137  (32.4%) | 112  (26.5%) | 90  (21.3%) | 51  (12.1%) | 33  (7.8%) |

**Table (6): Distribution of students' total Practice of AI(No=423).**

**Figure (2): Bar graph representing Participating Medical Students' Preferences Regarding AI Diagnosis.**

**Table (7): Correlation matrix of participants' knowledge, attitude, Practice**

|  | **Pearson correlation coefficient (r)** | | |
| --- | --- | --- | --- |
|  | **Knowledge** | **Attitude** | **Practice** |
| - Knowledge score - Attitude score - Practice level | 1.00  0.064  0.303* | 1.00  0.082 | 1.00 |

**** Correlation is significant***

p=<.001

***The table*** shows that there is statistically significant positive correlation between knowledge about AI and Practice (r = 0.303, p=<0.001), where Practice of AI tools increased with high knowledge about AI.

**Table (8): Binary logistic regression model for knowledge about AI**

| **Model** |  | | | | **95% C.I. for EXP(B)** | |
| --- | --- | --- | --- | --- | --- | --- |
|  | **B** | **S.E.** | **P- Value** | **OR** | **Lower** | **Upper** |
| Sex (Male) | .627 | .231 | .007* | 1.871 | 1.191 | 2.941 |
| Al-Azhar university |  |  | .003* |  |  |  |
| Alexandria University | -.211 | .440 | .632 | .810 | .342 | 1.917 |
| Beni Suef University | -.966 | .475 | .042* | .381 | .150 | .966 |
| Cairo University | .051 | .500 | .919 | 1.052 | .395 | 2.805 |
| Fayoum University | -.548 | .754 | .468 | .578 | .132 | 2.537 |
| Mansoura University | -.630 | .429 | .142 | .533 | .230 | 1.235 |
| Minia University | .070 | .511 | .892 | 1.072 | .394 | 2.918 |
| October 6 University | 1.267 | .582 | .029* | 3.551 | 1.135 | 11.111 |
| Suez Canal University | 1.794 | 1.096 | .102 | 6.016 | .702 | 51.568 |
| Zagazig University | .069 | .415 | .867 | 1.072 | .475 | 2.418 |
| Clinical Phase | -.619 | .306 | .043* | .539 | .296 | .981 |
| Residency (urban) | .188 | .250 | .451 | 1.207 | .740 | 1.970 |
| living situation? (Living with others) | .193 | .381 | .612 | 1.213 | .575 | 2.558 |
| Constant | .493 | .551 | .371 | 1.637 |  |  |

*R^2^= 0.143*

*Variable(s) entered on equation: sex, university, Academic or clinical phase, residency and living situation*

*Chi-square test for model coefficient =47.209, P-value < 0.001**

**Statistical significance*

**Inferential statistics of knowledge:**

**Table (9): Distribution of knowledge about generative AI in relation to gender**

| **Gender** | | **participants** | | |
| --- | --- | --- | --- | --- |
|  |  | **Yes N (%)** | **No N (%)** | **P Value** |
| **Do you have a solid knowledge of the basics of AI?** | **Male** | 98 (51%) | 94 (49%) | 0.033 |
|  | **Female** | 94 (40.7%) | 137 (59.3%) |  |
| **Do you know any application of AI in your field of interest** | **Male** | 140 (72.9%) | 52 (27.1%) | <0.001 |
|  | **Female** | 130 (56.3%) | 101 (43.7%) |  |
| **Familiar with the concept of AI in education ?** | **Male** | 148 (77.1%) | 44 (22.9%) | 0.005 |
|  | **Female** | 149 (64.5%) | 82 (35.5%) |  |
| **Familiar with the various AI tools available for educational purposes(Chat-gpt, Gemini, Bing, ……)?** | **Male** | 146 (76%) | 46 (24%) | 0.022 |
|  | **Female** | 152 (65.8%) | 79 (34.2%) |  |

*Chi-square test*

**Table (10): Distribution of knowledge about generative AI according to Academic Phase**

| **Academic Phase** | | **participants** | | |
| --- | --- | --- | --- | --- |
|  |  | **Yes N (%)** | **No N (%)** | **P Value** |
| **Do you have a solid knowledge of the basics of AI?** | **Phase 1**  **(Academic phase)** | 47  (60.3%) | 31 (39.7%) | 0.004 |
|  | **Phase 2**  **(clinical phase)** | 145 (42%) | 200 (58%) |  |
| **Do you know any application of AI in your field of interest** | **Phase 1**  **(Academic phase)** | 62 (79.5%) | 16 (20.5%) | 0.002 |
|  | **Phase 2**  **(clinical phase)** | 208 (60.3%) | 137 (39.7%) |  |
| **Have you ever been taught about AI in your under-graduate studies?** | **Phase 1**  **(Academic phase)** | 32 (41%) | 46 (59%) | 0.019 |
|  | **Phase 2**  **(clinical phase)** | 93 (27%) | 252 (73%) |  |
| **Does AI require a lot of labeled data to learn (data already processed by a human)?** | **Phase 1**  **(Academic phase)** | 58 (74.4%) | 24 (25.6%) | 0.027 |
|  | **Phase 2**  **(clinical phase)** | 209 (60.6%) | 136 (39.4%) |  |

*Chi-square test*

**Table (11): differences of knowledge about generative AI according to residency**

| **residency** | | **participants** | | |
| --- | --- | --- | --- | --- |
|  |  | **Yes N (%)** | **No**  **N (%)** | **P Value** |
| **Do you know any application of AI in your field of interest** | **Rural** | 81 (56.3%) | 63 (43.8%) | 0.025 |
|  | **Urban** | 189 (67.7%) | 90 (32.3%) |  |
| **Lack of time due to educational burden** | **Rural** | 49 (34%) | 95 (66%) | 0.014 |
|  | **Urban** | 63 (22.6%) | 216 (77.4%) |  |

*Chi-square test*

**Inferential statistics of Attitude:**

**Table (12): differences of attitude toward generative AI according to gender**

| **Gender** | | **Participants** | | | | | |
| --- | --- | --- | --- | --- | --- | --- | --- |
|  |  | **Strongly agree N (%)** | **Agree N (%)** | **Natural N (%)** | **Disagree N (%)** | **Strongly disagree N (%)** | **P Value** |
| **What is your general attitude towards the application of Al in medicine?** | **Male** | 25 (13%) | 71 (37%) | 70 (36.5%) | 24 (12.5%) | 2 (1%) | 0.038 |
|  | **Female** | 15 (6.5%) | 78 (33.8%) | 105 (45.5%) | 27 (11.7%) | 6 (2.6%) |  |
| **Only a health care professional can make the right decision about treatment and procedure.** | **Male** | 78 (40.6%) | 59 (30.7%) | 39 (20.3%) | 9 (4.7%) | 7 (3.6%) | 0.010 |
|  | **Female** | 56 (24.2%) | 104 (45%) | 41 (17.7%) | 25 (10.8%) | 5 (2.2%) |  |

*Mann-whitney U test*

**Table (13): differences of attitude toward generative AI according to Academic year**

| **Academic year** | | **Participants** | | | |
| --- | --- | --- | --- | --- | --- |
|  |  | **The Al almost never gives a false alarm but sometimes misses a diagnose)**  **N (%)** | **The Al give a false alarm about as often as it misses a diagnose,**  **N (%)** | **The Al misses almost no diagnosis but often gives a false alarm,**  **N (%)** | **P value** |
| **Suppose artificial intelligence makes a diagnosis, what would you prefer?** | **Phase 1 (Academic phase)** | 14  (17.9%) | 35 (44.9%) | 29 (37.2%) | 0.040 |
|  | **Phase 2 (Clinical phase)** | 96 (27.8%) | 153 (44.3%) | 96 (27.8%) |  |

*Chi-square test*

**Table (14): Attitude toward generative AI according to residency**

| **Residency** | | **Participants** | | | | | |
| --- | --- | --- | --- | --- | --- | --- | --- |
|  |  | **Strongly agree**  **N (%)** | **Agree**  **N (%)** | **Natural**  **N (%)** | **Disagree**  **N (%)** | **Strongly disagree**  **N (%)** | **P Value** |
| **I believe healthcare students should learn the basics of Al** | **Rural** | 31 (21.5%) | 67 (46.5%) | 40 (27.8%) | 4 (2.8%) | 2 (1.4%) | 0.004 |
|  | **Urban** | 97 (34.8%) | 119 (42.7%) | 50 (17.9%) | 9 (3.2%) | 4 (1.4%) |  |
| **I believe Al will revolutionize the educational system** | **Rural** | 21 (14.6%) | 61 (42.4%) | 46 (31.9%) | 12 (8.3%) | 4 (2.8%) | 0.032 |
|  | **Urban** | 50 (17.9%) | 140 (50.2%) | 68 (24.4%) | 18 (6.5%) | 3 (1.1%) |  |
| **I believe some specialties are more prone to be replaced by Al than others** | **Rural** | 8 (5.6%) | 53 (36.8%) | 41 (28.5%) | 36 (25%) | 6 (4.2%) | 0.004 |
|  | **Urban** | 53 (19%) | 102 (36.6%) | 56 (20.1%) | 53 (19%) | 15 (5.4%) |  |
| **Residency** | | **The Al almost never gives a false alarm but sometimes misses a diagnose)**  **N (%)** | **The Al give a false alarm about as often as it misses a diagnose**  **N (%)** | **The Al misses almost no diagnosis but often gives a false alarm**  **N (%)** |  |  | **P value** |
| **Suppose artificial intelligence makes a diagnosis, what would you prefer?** | **Rural** | 48 (33.3%) | 63 (43.8%) | 33 (22.9%) |  |  | 0.006 |
|  | **Urban** | 62 (22.2%) | 125 (44.8%) | 92 (33%) |  |  |  |

*Mann-whitney U test and chi-squre test*

**Inferential statistics of Practice:**

**Table (15): differences of practice of generative AI according to gender**

| **Gender** | | **Participants** | | | | | |
| --- | --- | --- | --- | --- | --- | --- | --- |
|  |  | **All the time**  **N (%)** | **Most of the time**  **N (%)** | **Often**  **N (%)** | **Rarely**  **N (%)** | **Never**  **N (%)** | **P Value** |
| **How frequently do you use Al to prepare for your exams?** | **Male** | 6 (3.1%) | 19 (9.9%) | 61 (31.8%) | 68 (35.4%) | 38 (19,8%) | <.001 |
|  | **Female** | 7 (3%) | 16 (6.9%) | 52 (22.5%) | 69 (29.9%) | 87 (37.7%) |  |
| **How frequently do you use Al to conduct your research?** | **Male** | 21 (10.9%) | 41 (21.4%) | 60 (31.3%) | 38 (19.8%) | 32 (16.7%) | <.001 |
|  | **Female** | 11 (4.8%) | 35 (15.2%) | 70 (30.3%) | 52 (22.5%) | 63 (27.3%) |  |
| **How frequently do you use Al to prepare for your homework/assignment?** | **Male** | 13 (6.8%) | 28 (14.6%) | 59 (30.7%) | 55 (28.6%) | 37 (19.3%) | 0.021 |
|  | **Female** | 7 (3%) | 31 (13.4%) | 64 (27.7%) | 61 (26.4%) | 68 (29.4%) |  |

*Mann-whitney U test*

**Table (16): differences of practice of generative AI according to Academic year**

| **Academic year** | | **Participants** | | | | | |
| --- | --- | --- | --- | --- | --- | --- | --- |
|  |  | **All the time**  **N (%)** | **Most of the time**  **N (%)** | **Often**  **N (%)** | **Rarely**  **N (%)** | **Never**  **N (%)** | **P Value** |
| **How frequently do you use Al to prepare for your homework /assignment?** | **Phase 1 (Academic phase)** | 5 (6.4%) | 18 (23.1%) | 25 (32.1%) | 15 (19.2%) | 15 (19.2%) | 0.006 |
|  | **Phase 2 (Clinical phase)** | 15 (4.3%) | 41 (11.9%) | 98 (28.4%) | 101 (29.3%) | 90 (26.1%) |  |

*Mann-whitney U test*

**Table (17): differences of practice of generative AI according to residency**

| **Residency** | | **Participants** | | | | | |
| --- | --- | --- | --- | --- | --- | --- | --- |
|  |  | **All the time**  **N (%)** | **Most of the time**  **N (%)** | **Often**  **N (%)** | **Rarely**  **N (%)** | **Never**  **N (%)** | **P Value** |
| **How frequently do you use Al to prepare for your exams?** | **Rural** | 7 (4.9%) | 10 (6.9%) | 43 (29.9%) | 54 (37.5%) | 30 (20.8%) | 0.035 |
|  | **Urban** | 6 (2.2%) | 25 (9%) | 70 (25.1) | 83 (29.7) | 95 (34.1%) |  |
| **How frequently do you use AI for personality development and other skills, like courses?** | **Rural** | 13 (9%) | 18 (12.5%) | 38 (26.4%) | 44 (30.6) | 31 (21.5%) | 0.006 |
|  | **Urban** | 20 (7.2%) | 33 (11.8%) | 52 (18.6%) | 68 (24.4%) | 106 (38%) |  |

*Mann-whitney U test*
